# Supplementary figures and images for: Network analysis of depression and anxiety symptoms in Chinese rheumatoid arthritis patients
Source: PeerJ. 2023 Nov 6;11:e16356. doi: 10.7717/peerj.16356 (PMC10634336; doi:10.7717/peerj.16356)

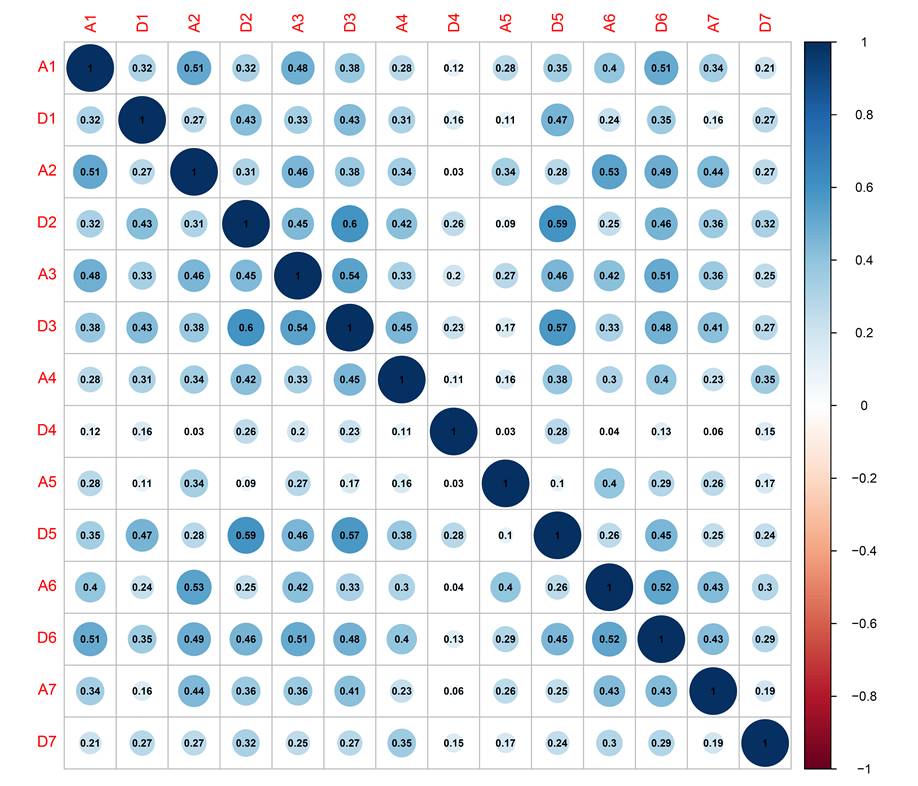

Supplement: Supplemental Information 2 [file peerj-11-16356-s002.jpg]

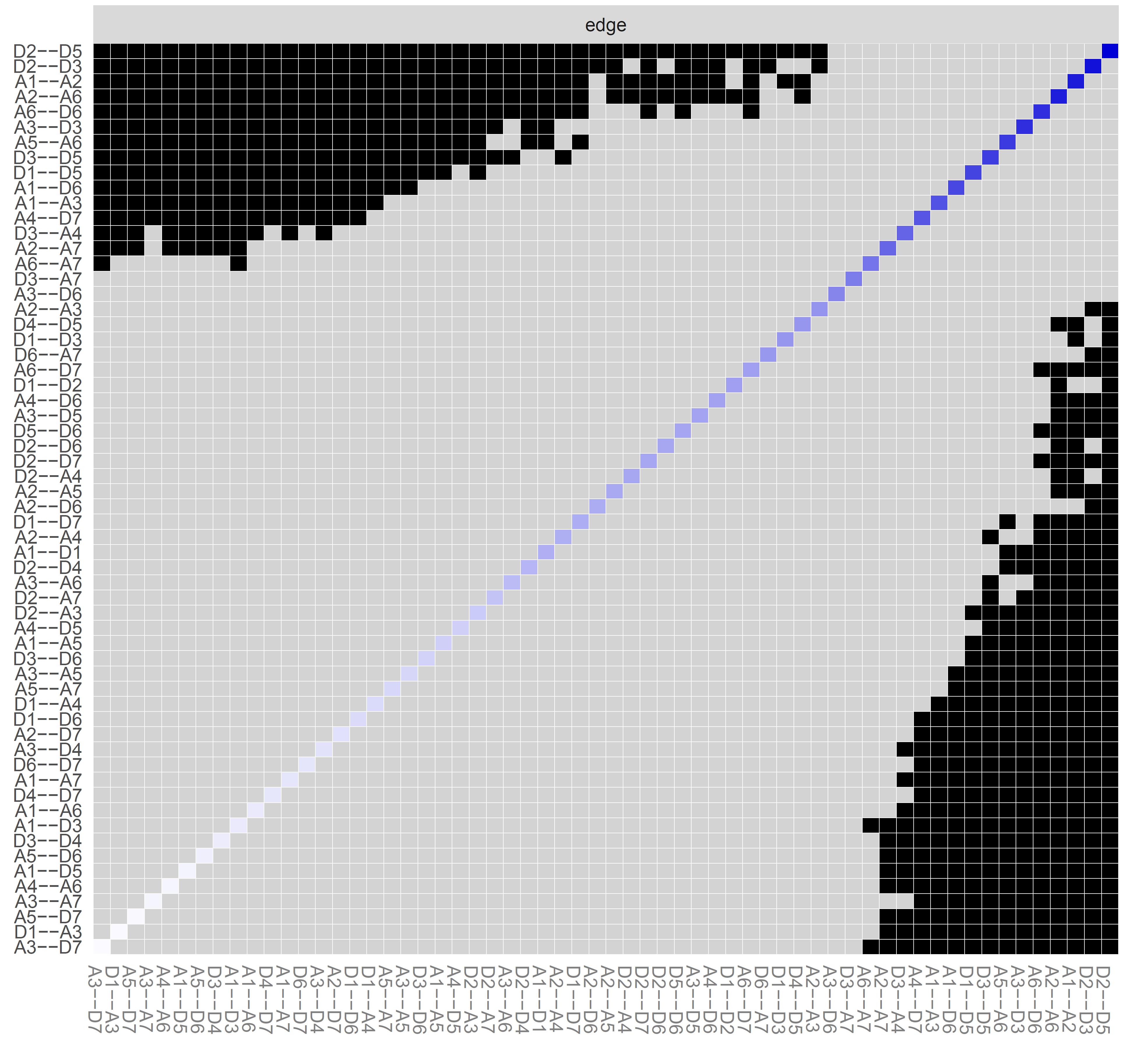

Supplement: Supplemental Information 3 [file peerj-11-16356-s003.jpg]

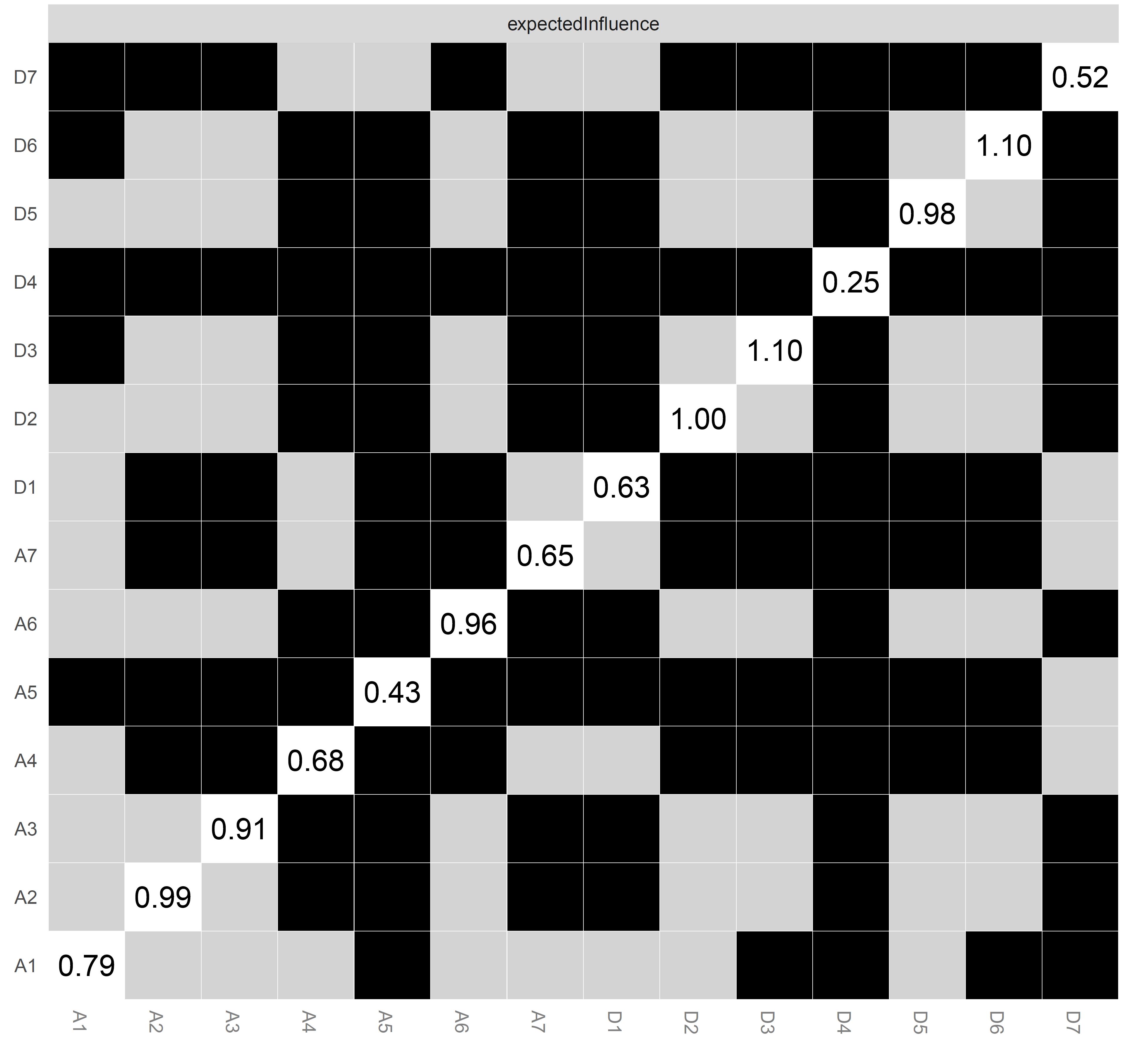

Supplement: Supplemental Information 4 [file peerj-11-16356-s004.jpg]

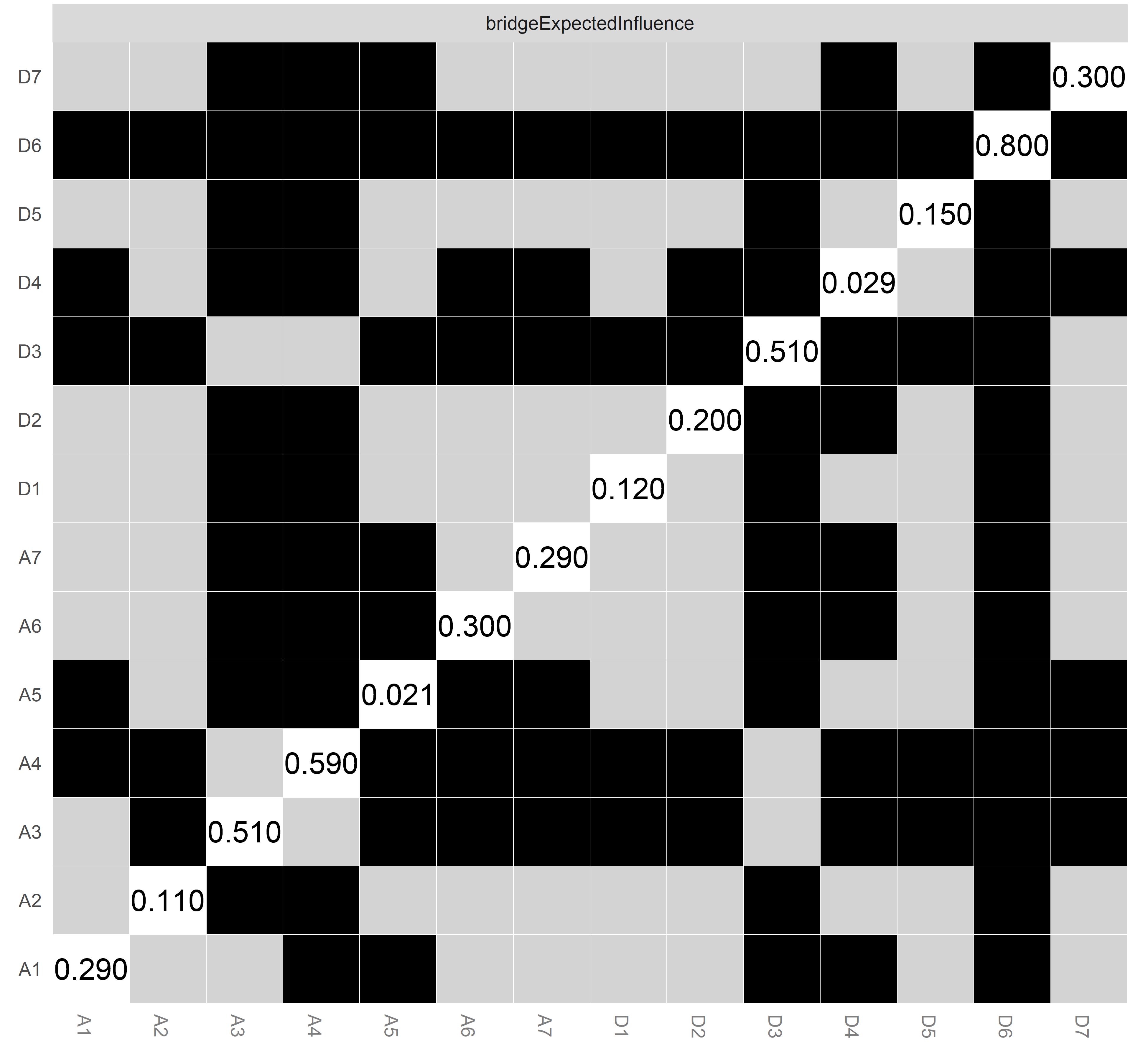

Supplement: Supplemental Information 5 [file peerj-11-16356-s005.jpg]
